# Altered levels of IFN-γ, IL-4, and IL-5 depend on the *TLR4* rs4986790 genotype in COPD smokers but not those exposed to biomass-burning smoke

**DOI:** 10.3389/fimmu.2024.1411408

**Published:** 2024-07-30

**Authors:** Karla Jazmín Gutiérrez-Romero, Ramcés Falfán-Valencia, Alejandra Ramírez-Venegas, Rafael De Jesus Hernández-Zenteno, Fernando Flores-Trujillo, Raúl Sansores-Martínez, Espiridión Ramos-Martínez, Gloria Pérez-Rubio

**Affiliations:** ^1^ HLA Laboratory, Instituto Nacional de Enfermedades Respiratorias Ismael Cosío Villegas, Mexico City, Mexico; ^2^ Tobacco Smoking and COPD Research Department, Instituto Nacional de Enfermedades Respiratorias Ismael Cosío Villegas, Mexico City, Mexico; ^3^ Clínica de Enfermedades Respiratorias, Fundación Médica Sur, Mexico City, Mexico; ^4^ Experimental Medicine Research Unit, Facultad de Medicina, Universidad Nacional Autónoma de México, Mexico City, Mexico

**Keywords:** COPD, tobacco smoke, biomass-burning smoke, SNPs, cytokines

## Abstract

**Introduction:**

Chronic obstructive pulmonary disease (COPD) is associated with tobacco smoking and biomass-burning smoke exposure. Toll-like receptor 4 (*TLR4*) single-nucleotide polymorphisms (SNPs) may contribute to its pathogenesis. The study aimed to assess the association of rs4986790 and rs4986791 in the *TLR4* gene in a Mexican mestizo population with COPD secondary to tobacco smoking (COPD-TS) and biomass-burning smoke (COPD-BBS) and to evaluate whether the genotypes of risk affect cytokine serum levels.

**Materials and methods:**

We enrolled 2,092 participants and divided them into two comparisons according to their environmental exposure. SNPs were genotyped using TaqMan probes. Serum cytokine levels (IL-4, IL-5, IL-6, IL-10, and INF-γ) were quantified by ELISA.

**Results:**

The rs4986790 AA genotype in COPD-TS was associated with a higher COPD risk (OR = 3.53). Haplotype analysis confirmed this association, identifying a block containing the rs4986790 allele (A–C, OR = 3.11). COPD-TS exhibited elevated IL-6, IL-4, and IL-5 levels compared with smokers without COPD (SWOC), whereas COPD-BBS displayed higher IFN-γ, IL-6, and IL-10 levels. The AA carriers in the COPD-TS group had elevated IL-4, IL-5, and IFN-γ compared with carriers of AG or GG.

**Conclusion:**

The rs4986790 common allele and the A–C haplotype (rs4986790–rs4986791) were associated with a higher COPD risk in smokers; COPD patients carrying the AA genotype showed increased pro-inflammatory cytokines.

## 1 Introduction

Chronic obstructive pulmonary disease (COPD) ranks third in terms of mortality, finding a higher prevalence in adults over 40 years old (1). Tobacco smoking accounts for over 70% of COPD cases in high-income countries; however, household air pollution is another risk factor (2). Around 2.3 billion people worldwide cook using open fires or inefficient stoves fueled by biomass (wood, animal dung, and crop waste), which generates harmful household air pollution (3).

In Mexico, according to the PLATINO (Spanish acronym for the Latin American Project for Research in Pulmonary Obstruction) study, the prevalence of COPD is 7.8% in people over 40 years of age. There is a direct relationship between COPD and the number of packs of cigarettes smoked; the risk of suffering from it increases if there is the consumption of more than 10 packs per year by 15.7% (4). In rural and suburban areas of Mexico, approximately 14.5 million people use firewood for cooking, with women being the most affected. The Institute National of Respiratory Diseases Ismael Cosío Villegas (INER) in Mexico City indicates that 30% of people who suffer from COPD are due to exposure to wood smoke; of these, 88% are women (5).

COPD is a complex and heterogeneous lung condition characterized by an atypical inflammatory response due to chronic exposure to noxious particles or gases and gene–environment interactions, leading to airflow obstruction (6, 7). The main environmental risk factor for the development of COPD is tobacco smoking; however, several meta-analyses support the association of biomass-burning smoke with COPD (8–10).

Studies on COPD by exposure to biomass smoke are scarce. Patients with COPD moderate to severe show high levels of Th1 cytokines and chemokines (11). Increased Th17 cells in serum and airway (12) have been reported. In addition, IL-4 and Th2 cells are elevated in patients with eosinophilic COPD (13). However, variants in *TLR4* and COPD by exposure to biomass have not been explored.

Toll-like receptors (TLRs) have crucial roles in initiating innate immune responses; to date, there are 13 types (14, 15). Multiple studies have demonstrated the importance of TLRs in developing the inflammatory response in COPD, predominantly TLR2, TLR4, and TLR9 (1, 16). TLR4 principally recognizes lipopolysaccharide (LPS) from Gram-negative bacteria and endogenous molecules from cell death caused by oxidative stress (1, 17). Cigarette smoke and air components, such as airborne particulate matter, can activate TLR4 (18, 19), initiating signal transduction pathways that culminate in the activation of transcription factors that regulate chemokine and cytokine expression, leading to the production of IL-1β, IL-6, IL-8, IL-10, TNF-α, and IFNs (20–22). The serum proteomic profile of patients with COPD shows that impaired innate immune response contributes to the development of the disease (23). Alveolar macrophages of COPD patients have M2 polarization characterized by the production of Th2 cytokines (24).

Previous research has evaluated the potential role of single-nucleotide polymorphisms (SNPs) in *TLR4* as a susceptibility factor implicated in distinct pathologies. For instance, rs4986790 (D299G) and rs4986791 (T399I) have been associated with decreased responsiveness and high susceptibility to different diseases (25–27). A murine model determined that the presence of alleles G and T (rs4986790 and rs4986791, respectively) in TLR4 produces hyporesponsiveness to LPS, reduced TLR4 surface expression *in vitro*, and increased sensitivity to Gram-negative and respiratory syncytial virus (RSV) infection (27). In this context, the role of these SNPs in the pathogenesis of COPD has been previously studied; still, the results are unclear and inconclusive. In Greek COPD patients, the T allele (rs4986791) was associated with an increased risk for the development of COPD in smokers (28). Analysis of rs4986790 in the Caucasian population showed a low frequency of the G allele in COPD non-smoker patients (29); however, in COPD patients with a smoking history of ≥10 pack years, there was no significant association (30). The allele G of rs4986790 modifies the extracellular domain of the TLR4 receptor; these structural changes influence the binding of ligands in region D299G, which leads to decreased ligand recognition and binding (31). *In vitro* studies suggest that the Asp299Gly/Thr399Ile haplotype affects phenotype, reducing cytokine production by the innate immune response (32). Therefore, this study aimed to evaluate the association of rs4986790 and rs4986791 in *TLR4* in a Mexican mestizo population with COPD secondary to tobacco smoking (COPD-TS) and secondary to biomass-burning smoke exposure (COPD-BBS) and to evaluate whether the genotypes of risk affect cytokine serum levels.

## 2 Materials and methods

### 2.1 Study population

We included 2,092 Mexican mestizo, unrelated subjects over 40 years of age and of indistinct sex, exposed to environmental risk factors; however, not all presented the disease; this group was the reference group because our study aimed to identify genetic variants associated with the risk of COPD. Participants were divided into two comparison groups based on the environmental risk factors associated with the development of COPD, and the analysis was done independently.

The tobacco smoking group included 379 subjects with COPD secondary to tobacco smoking (COPD-TS) and 1,073 smokers without COPD (SWOC); the criteria inclusion for this group were ≥5 cigarettes per day (cpd) at least 10 years and no history of exposure to biomass-burning smoke. The second comparison group included 180 subjects with COPD secondary to biomass-burning smoke (COPD-BBS) and 460 subjects with chronic exposure to biomass-burning smoke without COPD (BBES) as controls; the criteria of inclusion were exposure index to biomass-burning smoke (BEI) ≥100 h/year and subjects who were never smokers.

All the subjects with COPD were diagnosed in the Tobacco Smoking and COPD Research Department of the INER in Mexico City. The diagnosis of COPD was confirmed through spirometry from the FEV1/FVC ratio <70% after the bronchodilator administration, taking as reference the values for Mexicans (33).

All participants signed informed consent and received a privacy statement describing personal data protection. The protocol was approved by the INER Research and Ethics Committee in Mexico City (B03-23).

### 2.2 Blood samples and DNA isolation

From each participant, 7 mL of peripheral blood samples was drawn in an EDTA tube as an anticoagulant. DNA was extracted using the BDtractTM kit (Maxim Biotech Inc., San Francisco, CA, USA) and hydrated in TE buffer (Ambion, Waltham, MA, USA). The DNA was quantified by ultraviolet/visible light spectrophotometry using a NanoDrop 2000 spectrophotometer device (Thermo Scientific, Wilmington, DE, USA), considering the 260/280 and 260/230 ratio to evaluate the purity of the sample (1.8–2.0 and >2, respectively). Each sample was adjusted to 15 ng/µL for subsequent genotyping.

### 2.3 Genotyping

The SNPs were genotyped by real-time PCR employing a StepOnePlus Real-Time PCR System (Applied Biosystems, Foster City, CA, USA) by allelic discrimination through predesigned TaqMan probes for the rs4986790 (C:11722238_20) and rs4986791 (C:11722237_20) (Applied Biosystems, Foster City, CA, USA). The amplification reaction was performed in MicroAmp^®^ Optical 96-well reaction plates (Applied Biosystems; Woolston, UK), which included 3 µL of adjusted DNA per subject. Four non-template controls (NTC) were included as negative controls; 5% of the sample was genotyped by duplicate-like allelic designation control.

### 2.4 Cytokine level measurement

We selected 40 participants from each study group according to genotype in rs4986790. Circulating cytokines in the serum were quantified through ELISA. We determined the concentrations of IL-6 (PeproTech™, 900-M16) and IFN-γ (PeproTech™, 900-K27), two of the most representative cytokines of the Th1 immune response; IL-4 (PeproTech™, 900-M14) and IL-5 (PeproTech™, 900-M15), the central cytokines of the Th2 immune response; and IL-10 (PeproTech™, 900-M21), the most crucial regulatory cytokine. All procedures were conducted following the manufacturer’s recommendations. The capture antibody was diluted to 100 μg/mL in PBS, and 100 µL per well was placed in an ELISA plate (Nunc MaxiSorp™) and incubated overnight at room temperature. Subsequently, the wells were washed with 300 µL of washing solution (0.05% Tween 20 in PBS) per well. Next, 300 µL of blocking solution (1% BSA in PBS) was added). 100 µL of standard or serum sample was added to each well in duplicate and incubated at room temperature for 2 h, and then the plate was washed three times. The detection antibody was diluted to 0.25 μg/mL, and 100 µL was added per well and incubated at room temperature for 2 h. After this time, the plate was rewashed three times. Streptavidin-HRP was diluted at a 0.025-µg/mL concentration, and 100 µL was added per well. The plate was incubated for 30 min at room temperature. Subsequently, the plate was washed three times. 100 µL of the substrate solution was added to each well and incubated in the dark for development for 20 min. The colorimetric reaction was quantified in an ELISA plate; we read at 450 nm with wavelength correction set at 620 nm. Finally, according to the manufacturer’s recommendations, sample concentrations of cytokines were obtained by comparing them with a 7-point standard curve.

### 2.5 Statistical analysis

The sample size calculation was realized with the Latin American population’s minor allele frequency (MAF = 4.0) rs4986791. The odds ratio estimated was two. We included three controls for each case. The confidence interval was 95%, and the statistical power was 80%. With these criteria, we need 395 COPD and 1,185 controls in the tobacco-smoking group.

The demographic and clinical data were analyzed with SPSS version 26.0. The Kolmogorov–Smirnov test was used to evaluate the numerical data distributions. According to the distribution of the variables test, the Student’s T, or Mann–Whitney U test was performed. The categorical data were analyzed with the chi-square (χ^2^) and are presented as frequencies or percentages. The Hardy–Weinberg equilibrium (HWE) was calculated for both SNPs. Alleles and genotypes were analyzed using a contingency table using Epidat version 3.1 (34). We used genetics models and a logistic model age, sex, body mass index (BMI), and tobacco index (TI) or exposure index to biomass-burning smoke (BEI) were used as covariables in the genetic association, and we performed in Plink version 1.9 (35). Linkage disequilibrium (LD) analysis and haplotype association were performed in HaploView version 4.1 (36).

## 3 Results

### 3.1 Demographic and clinical data

We included 379 patients with COPD (COPD-TS) secondary to tobacco smoking and 1,073 smokers without COPD (SWOC). Significant differences exist in sex, age, and BMI. COPD-TS had a higher tobacco index than SWOC (40 vs. 21, respectively, p < 0.001). 46.9% of COPD-TS was GOLD 2 (moderate) (**Table 1**). A total of 180 patients with COPD secondary to biomass-burning smoke (COPD-BBS) and 460 subjects with chronic exposure to biomass-burning smoke without COPD (BBES) formed the control group. Most of the group comprised women (91.6% in COPD-BBS and 98.4% in BBES). The sex, age, and BMI were statistically significantly different. The biomass burning smoke exposure index (BEI) was higher in the COPD-BBS group than BBES (300 *vs*. 240 h per year, respectively, p = 0.003). 58% of patients with COPD by exposure to biomass-burning smoke was GOLD 2 (moderate) (**Table 1**).

**Table 1:** Demographic and clinical data of tobacco smoking and biomass-burning smoke groups.

Variable	COPD-TS (n = 379)	SWOC (n = 1,073)	*p-value*	COPD-BBS (n = 180)	BBES (n = 460)	*p-value*
Sex male, n (%)	270 (71.2)	478 (44.5)	<0.001^a^	15 (8.4)	7 (1.6)	<0.001^a^
Age (year)	63 (57–69)*	51 (44–58)	<0.001	72 (64–79)*	60 (54–70)	<0.001
BMI (kg/m^2^)	25.3 (22.4–28.6)	26.9 (24.3–29.7)	<0.001	26.2 (23.3–30.1)	28.3 (25.1–31.5)	<0.001
TI (packs/year)	40 (26–55)	21 (12–34)	<0.001	–	–	–
BEI (hours/year)	–	–	–	300 (180–400)	240 (152–353)	0.003
FVC (%)^b^	83 (68–98)	96 (87–105)	<0.001	86 (75–101)	97 (87–108)	<0.001
FEV1 (%)^b^	56 (40.5–73)	98 (89–108)	<0.001	66 (54.2–80.7)	103 (93–114)	<0.001
FEV1/FVC (%)^b^	53.5 (41–63)	81.9 (78–85.1)	<0.001	61 (51–66)	84 (79–88)	<0.001
GOLD, n (%) ^c^						
1	56 (15.0)	–	–	43 (26.2)	–	–
2	175 (46.9)	–	–	95 (58.0)	–	–
3	94 (25.2)	–	–	24 (14.6)	–	–
4	48 (12.8)	–	–	2 (1.2)	–	–

Data are expressed in median (quartile 25–75). The Mann–Whitney U test determined p values*. Diagnosis age; ^a^ p-value was determined by χ^²^ test; ^b^ data post-bronchodilator; ^c^ GOLD grades and severity of 2023 [2]. COPD-TS, subjects with COPD secondary to tobacco smoking; SWOC, smokers without COPD; COPD-BBS, subjects with COPD secondary to biomass-burning smoke; BBES, subjects with chronic exposure to biomass-burning smoke without COPD; BMI, body mass index; TI, Tobacco index; BEI, biomass burning smoke exposition index; FVC, forced vital capacity; FEV1, forced expiratory volume in the first second.

### 3.2 Genetic association analysis

For the comparison of COPD-TS and SWOC, the HWE was met in both genetic variants (p = 0.10 and p = 0.42 for rs4986790 and rs4986791, respectively). On the other hand, in comparing COPD-BBS and BBES, only rs4986791 accomplishes the HWE (p = 0.81), whereas rs4986790 does not (p = 0.003). Analysis of tobacco smoking groups showed statistically significant differences in both genetic variants (rs4986790 p = 2.08 × 10^−9^, rs4986791 p = 0.031); however, after performing a logistic regression to adjust for sex, age, BMI, and TI, only rs4986790 remained associated with decreased risk of developing COPD (p = 2.81 × 10^−5^, OR = 0.27, 95% CI = 0.17–0.43). We performed a full-genotype model for rs4986790 using sex, age, BMI, and TI as covariables; the AA genotype was associated with a high risk of COPD (p ≤ 0.001, OR = 3.53, 95% CI = 2.22–5.61). (**Table 2**).

**Table 2:** Allele and genotype analysis of tobacco smoking groups.

rs4986790	COPD-TS (n = 378)	SWOC (n = 844)	*p^c^*	OR (95% CI)
Alleles				
A	733 (97.0)	1,517 (89.9)	2.81 × 10^-5^	3.59 (2.30–5.60)
G	23 (3.0)	171 (10.1)		0.27 (0.17–0.43)
Full-genotype				
AA	356 (94.2)	686 (81.3)	<0.001	3.53 (2.22–5.61)
AG	21 (5.5)	145 (17.2)	<0.001	0.29 (0.18–0.47)
GG	1 (0.3)	13 (1.5)	0.100	0.17 (0.02–1.32)
rs4986791	COPD-TS (n = 345)	SWOC (n = 1,052)	*p*	OR (95% CI)
Alleles				
C	678 (98.3)	2034 (96.6)	0.044	1.94 (1.04-3.60)
T	12 (1.7)	70 (3.3)		0.51 (0.27–0.95)
Full-genotype				
CC	333 (96.5)	984 (93.5)	0.044	1.91 (1.02–3.58)
CT	12 (3.5)	66 (6.3)	0.067	0.53 (0.28–1.00)
TT	0 (0)	2 (0.2)	0.745	1.52 (0.13–16.83)

Data are expressed in numbers (%). p^c^ values were adjusted for age, sex, BMI, and TI. COPD-TS, patients with COPD secondary to tobacco smoking; SWOC, smokers without COPD; OR, odds ratio; CI, confidence interval.

The allele and genotype analysis for the biomass-burning smoke groups does not show statistically significant differences (**Supplementary Table S1**).

The patients of the COPD-TS and COPD-BBS groups were divided according to their GOLD stage to analyze the severity according to genotype. We considered GOLD 1 and 2 for a lower severity and GOLD 3 and 4 for a higher severity; however, regardless of risk factor exposure, we did not find a significant association (**Supplementary Tables S2 and S3**).

**Figure 1:**
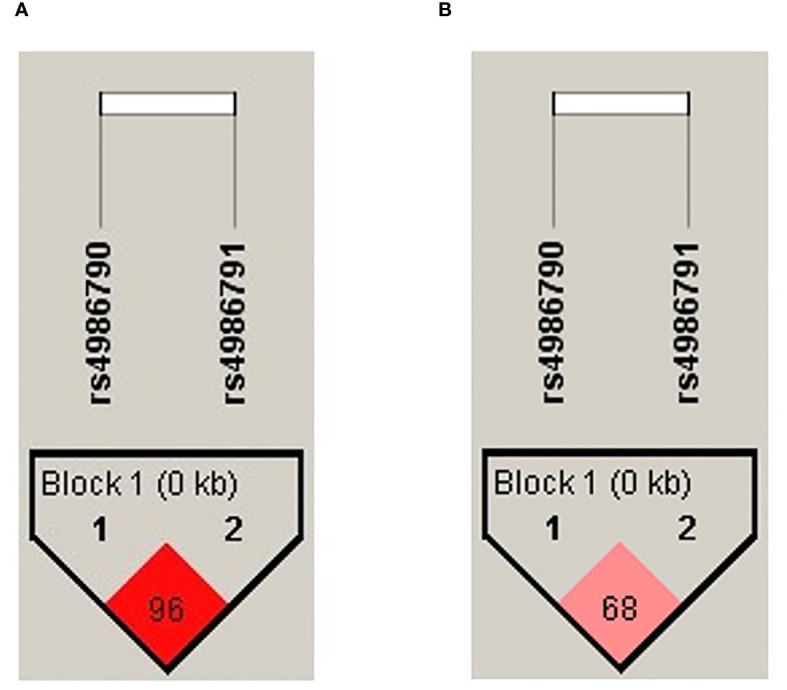
Haplotype block formed by the evaluated SNPs. The color intensity corresponds to the degree of linkage disequilibrium (LD); the D′ value is shown inside the block. **(A)** Haplotype plot for the tobacco-smoking group. **(B)** Haplotype plot for the biomass-burning smoke group.

The haplotype association for the tobacco smoking group was performed, and the formation of a block with high linkage disequilibrium (D′ = 0.96) between both SNP evaluated was observed (**Figure 1A**). For the biomass-burning smoke group, we observed a block formation with moderate LD (D′ = 0.68) (**Figure 1B**).

The haplotype analysis between COPD-TS and SWOC showed one block containing the common allele of rs4986790 associated with an increased risk of COPD (A–C, p = 9.13 × 10^−8^, OR = 3.11, 95% CI = 1.98–4.90). Two haplotypes were associated with decreased risk (G–C, p = 8.81 × 10^−7^, OR = 0.25, 95% CI = 0.13–0.47; G–T, p = 0.0295, OR = 0.46, 95% CI = 0.24–0.89). The analysis in the biomass-burning smoke group showed one haplotype with the common alleles of both SNP; however, we found no significant association (A–C, p = 0.08, OR = 0.49, CI = 0.22–1.10) (**Table 3**).

**Table 3:** Haplotype frequencies of rs4986790 and rs4986791.

Haplotype	COPD-TS (%)	SWOC (%)	*p-value*	OR (95% CI)
A–C	96.8	90.3	9.13 × 10^-8^	3.11 (1.98–4.90)
G–C	1.6	6.5	8.81 × 10^-7^	0.25 (0.13–0.47)
G–T	1.6	3.2	0.0295	0.46 (0.24–0.89)
	COPD-BBS (%)	BBES (%)	*p-value*	OR (95% CI)
A–C	96.8	98.4	0.08	0.49 (0.22–1.10)

COPD-TS, subjects with COPD secondary to tobacco smoking; SWOC, smokers without COPD; COPD-BBS, subjects with COPD secondary to biomass-burning smoke; BBES, subjects with chronic exposure to biomass-burning smoke without COPD; OR, Odds ratio; 95% CI, 95% confidence interval.

### 3.3 Analysis of cytokine levels in serum

**Figure 2:**
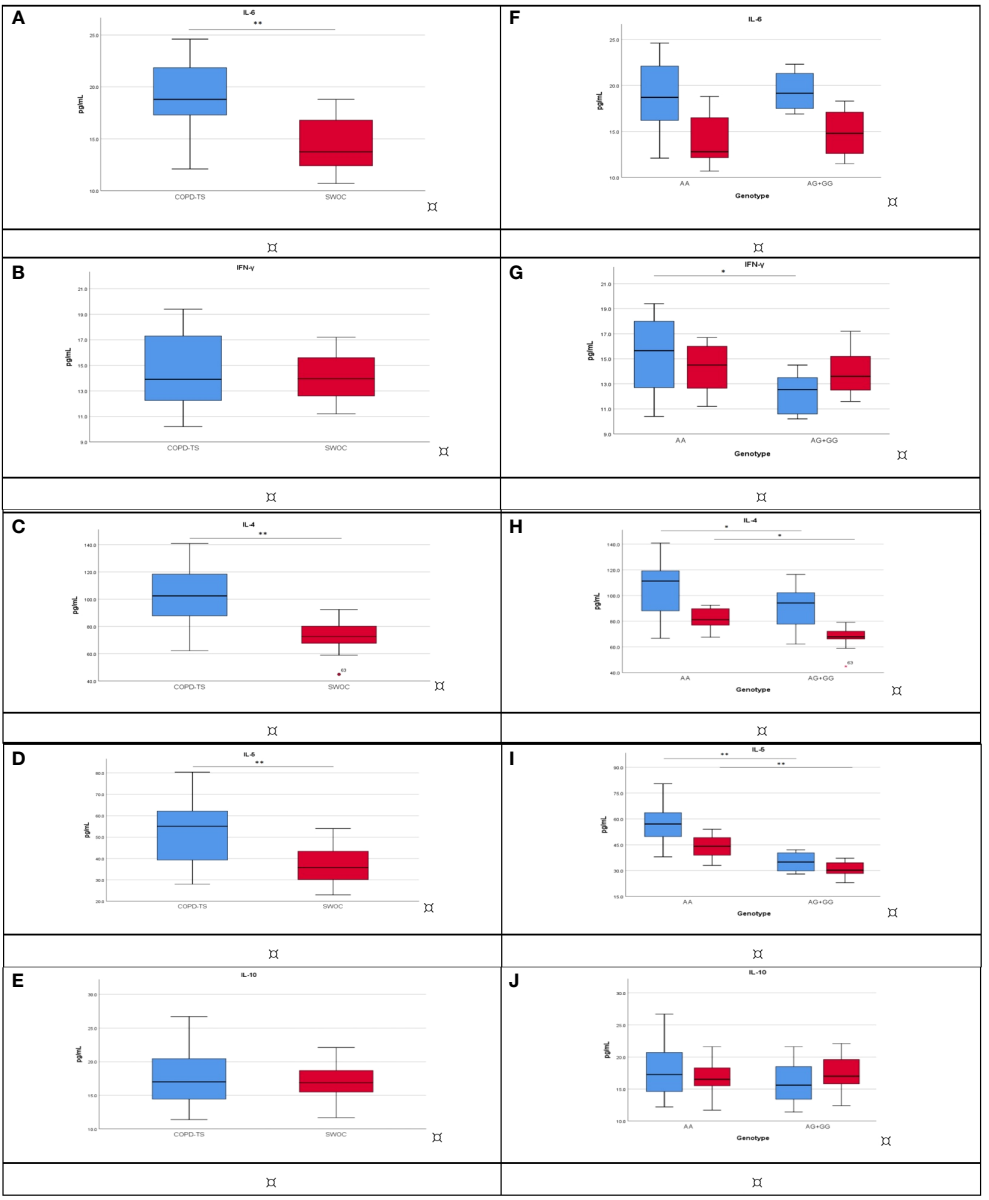
Cytokine levels compare COPD-TS (n = 40; blue) and SWOC (n = 40; red). The analysis was performed using the Mann–Whitney U or Student’s *t*-test, depending on the distribution. **p < 0.001; *p < 0.05. **(A)** IL-6; **(B)** IFN-γ; **(C)** IL-4; **(D)** IL-5; **(E)** IL-10. Association among cytokine levels and rs4986790 genotypes between COPD-TS [AA (n = 30), AG+GG (n = 10); blue] and SWOC [AA (n = 19), AG+GG (n = 21); red]. The analysis was performed using the Kruskal–Wallis test, and corrections were made for multiple tests using the Bonferroni method. *p < 0.05; **p < 0.001, Bonferroni *p* values are presented. **(F)** IL-6; **(G)** IFN-γ; **(H)** IL-4; **(I)** IL-5; **(J)** IL-10. COPD-TS, subjects with COPD secondary to tobacco smoking; SWOC, smokers without COPD.

We included 40 participants from each group. The demographic and clinical data are shown in the supplementary material (**Supplementary Table S4**). In the tobacco smoking group, significant differences were found in age (p < 0.001), BMI (p = 0.003), and TI (p = 0.023). Meanwhile, in the biomass-burning smoke group, significant differences were found in the sex (p = 0.001) (**Supplementary Table S4**). The COPD-TS showed high levels of IL-6 (18.9 pg/mL), IL-4 (102.9 pg/mL), and IL-5 (55.1 pg/mL) in comparison with SWOC (13.7 pg/mL, 74.5 pg/mL, and 35.7 pg/mL, respectively, p < 0.001) (**Figures 2A, C, D**; **Supplementary Table S5**). We did not find significant differences in IFN-γ and IL-10 (**Figures 2B, E**; **Supplementary Table S5**). In the case of the exposure to biomass-burning smoke groups, in the comparison of cytokine levels between COPD-BBS and BBES, a significant difference was obtained (p < 0.001) between the cytokine levels of IL-6 (28.3 pg/mL *vs*. 19.5 pg/mL), IFN-γ (35.6 pg/mL *vs*. 17.6 pg/mL), IL-4 (28.6 pg/mL *vs*. 20.2 pg/mL), and IL-5 (48.5 pg/mL *vs*. 29.4 pg/mL). Levels of IL-10 do not show significant differences (**Supplementary Figures 1A–D**).

We compared cytokine levels among COPD patients; the serum of COPD-TS has higher levels of IL-4 in comparison with COPD-BBS (102.9 pg/μL vs. 28.7 pg/μL) and the same to IFN-γ (13.9 pg/μL vs. 36.7 pg/μL, respectively); however, COPD-BBS has higher levels of IL-6 and IL-10 concerning COPD-TS (**Table 4**).

**Table 4:** Cytokine level comparison between COPD-TS and COPD-BBS.

Cytokine (pg/mL)	COPD-TS (n = 40)	COPD-BBS (n = 40)	p*
IL-4	102.9 (± 21.1)	28.7 (± 4.8)	<0.001^a^
IL-5	55.1 (39.2–62.3)	48.5 (38.8–53.05)	0.059
IL-6	18.9 (17.3–22.0)	28.35 (23.9–32.1)	<0.001
IL-10	17.0 (14.4–20.5)	20.8 (19.6–24.0)	<0.001
IFN-γ	13.9 (12.1–17.3)	36.5 (29.6–41.9)	<0.001

Data are expressed in mean (± standard deviation) or median (quartile 25–75). The Mann–Whitney U test determined P-values; ^a^ p-value was determined by Student’s t-test. *Bonferroni correction. COPD-TS, subjects with COPD secondary to tobacco smoking; COPD-BBS, subjects with COPD secondary to biomass-burning smoke.

Having found an association for rs4986790 in smokers with COPD, we analyzed depending on their genotype (AA *vs*. AG or GG). IL-6 did not differ statically significantly (**Figure 2F**). Significant differences (p = 0.033) were observed in COPD-TS carriers to AA, who had high IFN-γ levels compared with COPD-TS carriers of AG or GG (15.6 pg/mL vs. 12.5 pg/mL). There are no differences in SWOC groups (**Figure 2G**). IL-4 showed significant differences in the comparison with the carrier of genotype AA vs. AG or GG for patients with COPD (111.2 pg/mL *vs*. 94.2 pg/mL), and the same trend was observed in SWOC group AA vs. AG+GG (81.2 pg/mL *vs*. 67.8 pg/mL; p = 0.035) (**Figure 2H**). Patients with COPD and carriers of the AA genotype had higher levels of IL-5 in comparison with the AG or GG genotype (57.0 pg/mL *vs*. 34.9 pg/mL, p < 0.001), and the SWOC group carrier of the AA genotype showed high levels of IL-5 compared with AG or GG (44.1 pg/mL *vs*. 30.2 pg/mL) (**Figure 2I**). Finally, IL-10 did not have significant differences (**Figure 2J**, **Supplementary Table S6**). In the case of the exposure to biomass-burning smoke groups, the analysis of levels of cytokines by genotype in rs4986790, we did not find statistically significant differences (**Supplementary Table S6**).

## 4 Discussion

COPD is a complex and multifactorial lung condition whose main environmental risk factors are tobacco smoking and exposure to biomass-burning smoke. However, other factors influence COPD development (37, 38). Genetic factors are also significant risk factors that might explain the disease, prognosis, exacerbations, and even response to therapy (37).

TLR4 in lung tissue may be activated by risk factors such as cigarette smoking, inhaled polluted air, and infection of bacteria and viruses (1). It upregulates the expression of inflammatory mediators, which induces alveolar macrophages to produce many inflammatory cytokines. This mechanism causes the neutrophils, CD^8+^ T lymphocytes, and other cells to participate in the inflammatory response of COPD (39).

In the present study, we evaluate the association between two SNPs in *TLR4* and COPD between two comparison groups exposed to the two main risk factors in the Mexican population: tobacco smoking and biomass-burning smoke. In Mexico, the smoking prevalence in 2020 was 7.2% in women and 26.3% in men, representing a public health problem mainly in urban areas (40). On the other hand, it is estimated that 20% of the population uses biomass as a principal energy source, mainly in low-income and rural areas (41).

In the tobacco-smoking groups, we found statistically significant differences in sex and predominance of men in COPD-TS in comparison with SWOC. Patients with COPD are older than SWOC (63 vs. 51 years), and the same trend for TI (40 vs. 21 packs/years). 46.9% of COPD-TS were GOLD 2 followed by GOLD 3 (25.2%). On the other hand, the biomass-burning smoke group was mainly composed of women (>90% in both patients’ groups). Exposure to biomass-burning smoke has been reported to be an important environmental risk factor primarily associated with women using biomass as a cooking fuel (42). More than half of the patients had GOLD 2, following GOLD 1. Due to these differences, logistic regression was performed to adjust for sex, age, BMI, and TI or BEI in the genetic association analysis. The primary ligand of TLR4 is LPS, the main component of Gram-negative bacterial cell walls. It has been shown that both the LPS contained in cigarette smoke, in addition to damage-associated molecular patterns (DAMPS) released by the interaction of epithelial cells with the combustion products of cigarette or biomass-burning smoke, can activate the TLR4 pathway and contribute to amplified inflammatory mechanisms in the lung (1, 19, 43). Two non-synonymous SNPs in the *TLR4* gene have been previously described, an A896G transition (rs4986790) and a C1196T transition (rs4986791) (27, 44), which have been shown to cause LPS hyporesponsiveness (44). These SNPs have been associated with different infectious diseases; for example, the GG genotype of rs4986790 has been associated with susceptibility to infection by *Helicobacter pylori* (45), or the amino acid change produced by these SNPs (Asp299Gly for rs4986790; Thr399Ile for rs4986791) results in reduced receptor function in infection by respiratory syncytial virus (46). Similarly, these SNPs have also been studied in non-infectious diseases, where the association of the rs4986790 G allele with a lower risk of developing atherosclerosis (47). However, a limited number of studies have described the role of these SNP in *TLR4* in the pathogenesis of COPD, and the reports focus on Caucasian populations.

To the best of our knowledge, this is the first study evaluating the association between rs4986790 and rs4986791 in COPD in the Mexican population. We found that the rs4986790 (allele A) is strongly associated (p = 2.81 × 10^−5^) with a high risk (OR = 3.59) of COPD secondary to tobacco smoking. The same risk association was found in the AA genotype and COPD (p ≤ 0.001, OR = 3.53). In a Chinese population, the AG (rs4986790) genotype was associated with a lower risk of COPD (OR = 0.32) (48). A Greek cohort found that individuals carrying the C allele (rs4986791) are at increased risk of COPD secondary to tobacco smoking (OR = 2.4) (28). Another study in a Greek population found that the rs4986790 G allele or the rs4986791 T allele was associated with the lower COPD stage (49). We did not find a significant association with any SNP evaluated when stratifying by the GOLD stage. Contradictory results exist; however, we must analyze the study design, ancestral contribution, phenotype of the disease, and smoking status (current or former smokers).

The haplotype analysis showed that the block with high LD (D′ = 0.96) formed by common alleles in both SNPs (A–C) is associated (p = 9.13 × 10^−8^) with the risk of COPD secondary to smoking (OR = 3.11). Our results indicate no association between the SNP evaluated and COPD secondary to biomass-burning smoke exposure. Likewise, there was no association depending on the GOLD stage. However, the association between COPD secondary to biomass-burning smoke and SNPs in genes related to innate immune response has been demonstrated in other studies. SNPs in genes of the HSP90 family (GC and CC genotype of rs2070908) were associated with a decreased risk of COPD secondary to biomass-burning smoke (OR = 0.6) (50). This family of genes codifies chaperone proteins that participate in protein folding and improve the detection and signaling related to TLR2, TLR4, and TLR9 (50, 51).

For LPS recognition, TLR4 forms a heterodimer with myeloid differentiation factor 2 (MD-2), allowing TLR4 activation (52). Once LPS recognition occurs, TLR4 oligomerizes and recruits downstream adaptors with the Toll-interleukin-1 receptor (TIR) domain (17). TLR4 can initiate two signaling pathways depending on the adaptor protein activated: the MyD88-dependent pathway, which results in the activation of transcription factor NF-kB and the production of proinflammatory cytokines, and the TRIF-dependent pathway, which results in the activation of transcription factor IRF3, responsible for the transcription of type 1 interferon, and NF-kB (53–55). The rs4986790 and rs4986791 have been associated with TLR4 hyporesponsiveness. In the airway, epithelial cells obtained from healthy Caucasian individuals, heterozygous for both SNPs, showed a reduced TLR4 response to LPS stimulation (25). In a murine model, both MyD88-dependent and TRIF-dependent signaling pathways are affected by the presence of homologous SNPs (D298F for rs4986790 and N397I for rs4986791), in addition to an increased risk of infections by Gram-negative microorganisms, influenza virus, and respiratory syncytial virus (27).

Systemic inflammation in COPD is characterized by immune response markers in peripheral blood, such as IL-6, with higher levels in COPD compared with smoking controls (56, 57). According to a meta-analysis, serum IL-6 concentrations in healthy individuals range from 4.6 pg/mL to 5.4 pg/mL (58). We showed in the SWOC group that levels of IL-6 were 13.7 pg/mL, and in patients with COPD-TS, these levels increased to 18.9 pg/mL. On the other hand, COPD-BBS had 1.4-fold levels of IL-6 compared with BBES (28.3 pg/mL *vs*. 19.5 pg/mL, respectively). Despite no significant differences in IL-6 levels, when stratified by genotype, both tobacco smoke and exposure to biomass-burning smoke subjects show a systemic inflammatory state.

On the other hand, patients with COPD and chronic bronchitis have inflammatory cells increased in the bronchial submucosal glands and mucosa of large airways. Gland-associated plasma cells express IL-4, which likely induces glycoprotein synthesis, promotes mucous cell hyperplasia, and, consequently, airway mucus secretion (59). IL-4 promotes macrophage activation and proliferation of fibroblasts and endothelial cells, increasing inflammatory cell recruitment to the lung (60). We have shown increased levels of IL-4 in COPD-TS and COPD-BBS. However, exclusively for COPD-TS, carriers of risk genotype (AA) in rs4986790 have higher values of IL-4 in comparison with patients with AG or GG.

In smokers with no obstructive chronic bronchitis, a high percentage of gland-associated plasma cells express the genes for IL-4 and IL-5 (59). The sputum of patients with COPD concentrations of IL-5 correlates with the degree of eosinophilia (61). We show in patients with COPD higher levels of IL-5. The COPD-TS group and carriers of the AA genotype in rs4986790 had higher levels of IL-5 for COPD-TS patients AG or GG genotype carriers. COPD presents distinct inflammatory phenotypes. While neutrophils, macrophages, and B-lymphocytes are the predominant inflammatory cell types in some patients, a significant proportion demonstrate airway eosinophilia (59).

IFN-γ has also been associated with the development of emphysema in mice (62). 75% of COPD patients producing IFN-γ from their sputum cells also released IFN-γ from their blood cells. The release of IFN-γ in COPD patients supported the role of the Th1 pathway at the local and systemic levels (63). We show high levels of IFN-γ in COPD-BBS concerning BBES. However, we did not find significant differences in the analysis by genotype. COPD-TS and SWOC had similar levels of IFN-γ, whereas in the study by genotypes, carriers of AA had higher levels of IFN-γ for AG or GG genotype. Despite its role in inhibiting several inflammatory pathways, we did not find differences in the IL-10 levels regardless of the risk factor for COPD, tobacco smoking, or smoke from burning biomass smoke (64).

TLRs participate in the adaptive immune response by activating T cells through antigen-presenting cells to induce effector and memory T-cell responses (55). CD^4+^ T cells differentiate into subsets of functional T-helper (Th) cells (Th1, Th2, Th17, Th22, Treg), where each subset is characterized by cytokine profiles (65, 66). Typically, Th1/Th2 cells maintain a relatively balanced state. However, different studies have indicated imbalances between Th1 and Th2 in various stages of COPD (67). COPD secondary to tobacco smoking has been typically associated with Th1/Th17 response (57).

High levels of Th2-associated cytokines in an acute exacerbation of COPD (AECOPD) compared with stable COPD suggest that COPD response can shift depending on the course of the disease (67). In addition, the inflammatory response may change depending on the environmental risk factor associated with COPD. Patients exposed to biomass-burning smoke present higher levels of IgE compared with tobacco smoke exposure, suggesting a Th2 response (68). A study in India found that the inflammatory response could vary depending on the biomass fuel used and exposure time, with both responses, Th1 and Th2, being possible (69).

**Figure 3:**
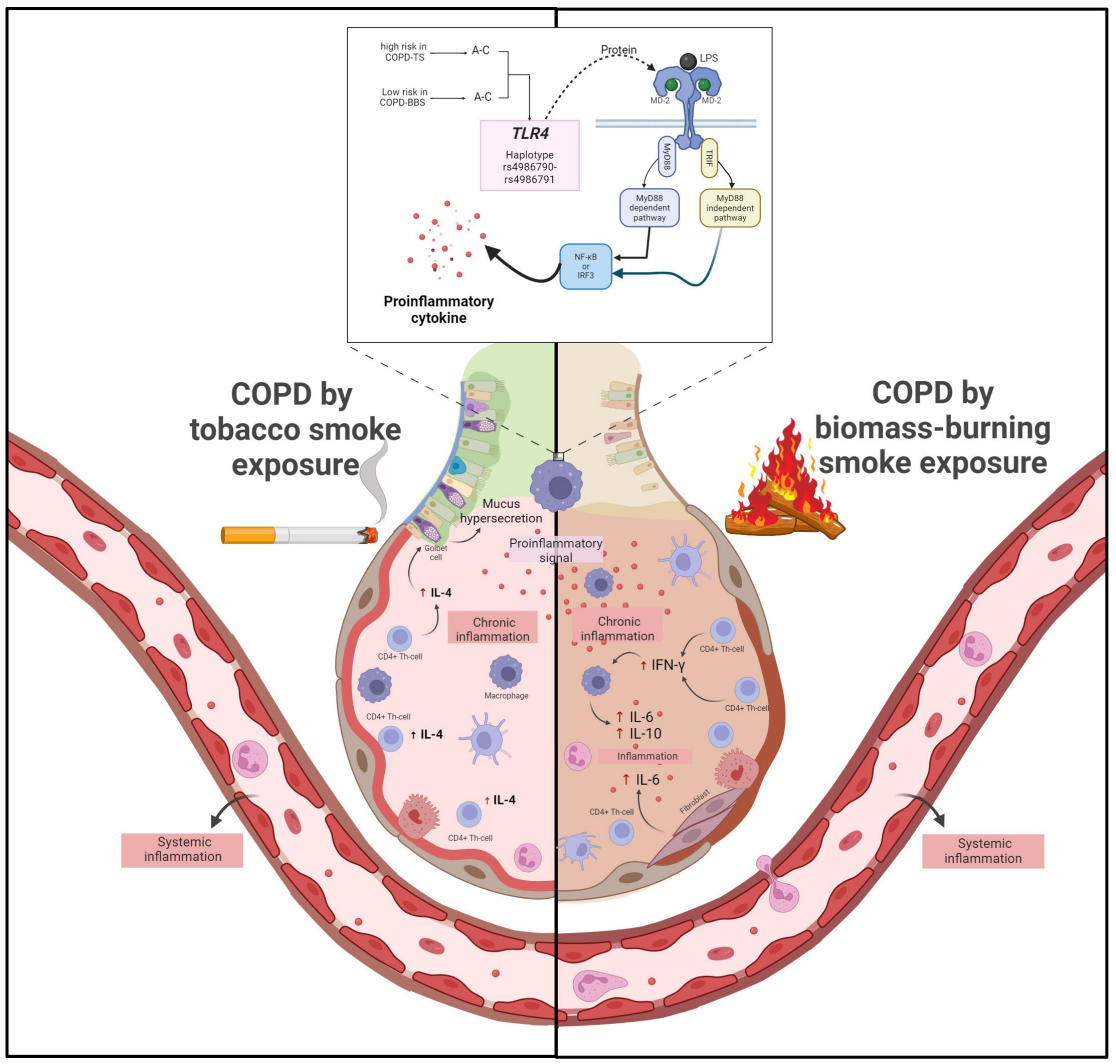
COPD secondary to tobacco smoking, pro-inflammatory signals from TLR4 activation trigger activation of CD^4+^ Th cells, which produce high levels of IL-4 that, in turn, cause mucus hypersecretion. On the other hand, in COPD, secondary to biomass burning, pro-inflammatory signals activate CD^4+^ Th1 cells, which produce high levels of IFN-γ. IFN-γ triggers the activation of macrophages, which deliver increased levels of IL-6 and fibroblasts, contributing to further inflammation. Additionally, in patients with COPD secondary to tobacco smoking, the AA risk genotype of rs4986790 causes an increase in IFN-γ, IL-4, and IL-5 levels, which exacerbate the inflammatory response.

Our study is not exempt from limitations; probably one was the sample size for the biomass-burning smoke group, which was considerably smaller than the tobacco-smoking group. In addition, only cytokines representative of each response were assessed. Our findings provide insights into added information about COPD pathogenesis depending on genetic contribution. This study contributes to knowledge and reinforces that COPD presents differences depending on the risk factors to which people are exposed. In COPD smokers, the rs4986790 AA directly affects the levels of IFN-γ, IL-4, and IL-5 that contribute to maintaining chronic inflammation (**Figure 3**).

In conclusion, the rs4986790 of TLR4 (A allele and AA genotype) and haplotype A–C (rs4986790–rs4986791) are associated with a high risk of COPD in smokers. Patients with COPD secondary to smoking have increased levels of IL-4 system; instead, patients with COPD secondary to biomass-burning smoke show high levels of IFN-γ, IL-6, and IL-10. The COPD smokers carrying the AA genotype (rs4986790, *TLR4*) showed high levels of IFN-γ, IL-4, and IL-5, which can contribute to maximizing chronic inflammation compared with patients’ carriers of genotypes AG or GG. Our study contributes to knowledge about the contribution of genetics in COPD and the phenotypes that differ according to exposure to risk factors for the disease.

## Data availability statement

The original contributions presented in the study are included in the article/Supplementary Materials. Further inquiries can be directed to the corresponding authors. Further data can also be found in the NCBI database, under the number: SUB13915964.

## Ethics statement

The studies involving humans were approved by INER Research and Ethics Committee in Mexico City. The studies were conducted in accordance with the local legislation and institutional requirements. The participants provided their written informed consent to participate in this study.

## Author contributions

KG-R: Writing – original draft, Formal analysis, Software, Investigation. RF-V: Writing – original draft, Funding acquisition, Supervision, Validation. AR-V: Supervision, Validation, Writing – review & editing. RH-Z: Writing – review & editing, Supervision, Visualization. FF-T: Supervision, Visualization, Writing – review & editing. RS-M: Supervision, Visualization, Writing – review & editing. ER-M: Conceptualization, Funding acquisition, Project administration, Software, Writing – original draft. GP: Conceptualization, Software, Writing – original draft, Formal analysis, Methodology.
