# Supplementary material for: Altered levels of IFN-γ, IL-4, and IL-5 depend on the TLR4 rs4986790 genotype in COPD smokers but not those exposed to biomass-burning smoke
Source: Front Immunol. 2024 Jul 30;15:1411408. doi: 10.3389/fimmu.2024.1411408 (PMC11319291; doi:10.3389/fimmu.2024.1411408)
Supplement: Supplementary file 1 [file DataSheet_1.docx]

Supplementary Material

**Table ST1**. Allele and genotype frequencies of biomass-burning smoke-exposed groups in *TLR4*.

| **rs4986790** | **COPD-BBS**  **(n=173)** | **BBES**  **(n=440)** | ***p*** | **OR (95%CI)** |
| --- | --- | --- | --- | --- |
| Alleles |  |  |  |  |
| A | 339 (98.0) | 867 (98.5) | 0.66 | 1.37 (0.54 - 3.48) |
| G | 7 (2.0) | 13 (1.5) |  |  |
| Codominant model |  |  |  |  |
| AA | 166 (96.0) | 428 (97.3) |  | 1 |
| AG | 7 (4.0) | 11 (2.5) | 0.49 | 0.64 (0.62 – 4.30) |
| GG | 0 (0) | 1 (0.2) |  |  |
| **rs4986791** | **COPD-BBS**  **(n=180)** | **BBES**  **(n=455)** | **p** | **OR (95% CI)** |
| Alleles |  |  |  |  |
| C | 353 (98.1) | 900 (98.9) | 0.36 | 1.78 (0.67 – 4.72) |
| T | 7 (1.9) | 10 (1.1) |  |  |
| Codominant model |  |  |  |  |
| CC | 173 (96.1) | 445 (97.8) |  | 1 |
| CT | 7 (3.9) | 10 (2.2) | 0.35 | 1.80 (0.67 – 4.80) |
| TT | 0 (0) | 0 (0) |  |  |

Abbreviations: COPD-BBS, patients with COPD secondary to biomass-burning smoke; BBES, subjects with chronic exposure to biomass-burning smoke without COPD; OR, Odds ratio; 95% CI, confidence interval.

**ST2.** Genotype frequencies according to GOLD stage of COPD-TS.

| **rs4986790** | **GOLD 1 + 2**  **(n=232)** | **GOLD 3 + 4**  **(n=142)** | ***p*** | **OR (95% CI)** |
| --- | --- | --- | --- | --- |
| AA | 221 (95.3) | 131 (92.3) |  |  |
| AG | 10 (4.3) | 11 (7.7) | 0.2 | 0.53 (0.22 – 1.30) |
| GG | 1 (0.4) | 0 (0) |  |  |
| **rs4986791** | **GOLD 1 + 2**  **(n=212)** | **GOLD 3 + 4**  **(n=131)** | ***p*** | **OR (95% CI)** |
| CC | 202 (95.3) | 129 (98.5) |  |  |
| CT | 10 (4.7) | 2 (1.5) | 0.2 | 0.31 (0.06 – 1.45) |
| TT | 0 (0) | 0 (0) |  |  |

Abbreviations: OR, Odds ratio; 95% CI, confidence interval.

**ST3.** Genotype frequencies according to the GOLD stage of COPD-BBS.

| **rs4986790** | **GOLD 1 + 2**  **(n=135)** | **GOLD 3 + 4**  **(n=26)** | ***p*** | **OR (95% CI)** |
| --- | --- | --- | --- | --- |
| AA | 131 (97.0) | 25 (96.1) |  |  |
| AG | 4 (3.0) | 1 (3.9) | 0.70 | 0.76 (0.08 – 7.11) |
| GG | 0 (0) | 0 (0) |  |  |
| **rs4986791** | **GOLD 1 + 2**  **(n=138)** | **GOLD 3 + 4**  **(n=26)** | ***p*** | **OR (95% CI)** |
| CC | 134 (97.1) | 26 (100) |  |  |
| CT | 4 (2.9) | 0 (0) | - | - |
| TT | 0 (0) | 0 (0) |  |  |

Abbreviations: OR, Odds ratio; CI, confidence interval

**ST4**. Demographic and clinical data of participants selected to evaluate cytokine levels in serum.

| **Variable** | **COPD-TS**  **(n=40)** | **SWOC**  **(n=40)** | ***p*** | **COPD-BBS**  **(n=40)** | **BBES**  **(n=40)** | ***p*** |
| --- | --- | --- | --- | --- | --- | --- |
| Sex male, n (%) | 30 (75) | 21 (52.5) | 0.06^a^ | 5 (12.5) | 0 (0) | 0.001^a^ |
| Age (year) | 62.65* (±5.55) | 58.5 (49.2 – 63) | <0.001^b^ | 67.45 (±6.03)* | 65 (±7.8) | 0.636 |
| BMI (kg/m^2^) | 24.55 (±4.13) | 27.56 (±4.76) | 0.003 | 28.5 (25.5 - 31.2) | 27.5 (25.1 – 32.7) | 0.695^b^ |
| TI (packs/year) | 40 (30 – 50) | 34.5 (20 - 45) | 0.023^b^ | - | - | - |
| BEI (h/y) | - | - | - | 300 (210 - 440) | 270 (185.5 – 328.5) | 0.073^b^ |
| Lung function data (post-bronchodilator) | | | | | | |
| FVC (%) | 82.13 (±21.93) | 99.19 (±16.63) | <0.001 | 87.71 (±19.43) | 96.63 (±20.46) | <0.001 |
| FEV1 (%) | 49.88 (±19.68) | 102.92 (±16.85) | <0.001 | 67.58 (±17.37) | 101.68 (±25.59) | 0.003 |
| FEV1/FVC (%) | 47.44 (±12.35) | 82.08 (±4.67) | <0.001 | 63 (52.6 – 66.6) | 84.3 (79 – 90) | <0.001^b^ |
| GOLD stage, n (%) | | | | | | |
| 1 | 4 (10) | - | - | 10 (25) | - | - |
| 2 | 15 (37.5) | - | - | 22 (55) | - | - |
| 3 | 15 (37.5) | - | - | 8 (20) | - | - |
| 4 | 6 (15) | - | - | - | - | - |

Data are expressed in mean (±standard deviation) and median (quartile 25 – 75). * Diagnosis age. *p* values were determined by Student’s *t*-test; ^a^ *p*-value was determined by χ^²^ test; ^b^ *p* value was determined by Mann-Whitney U test. Abbreviations: COPD-TS, subjects with COPD secondary to tobacco smoking; SWOC, smokers without COPD; COPD-BBS, subjects with COPD secondary to biomass-burning smoke; BBES, subjects with chronic exposure to biomass-burning smoke without COPD; BMI, body mass index; cpd, cigarette per year; TI, Tobacco index; BEI, biomass burning smoke exposition index; FVC, forced vital capacity; FEV1, forced expiratory volume in the 1^st^ second.

**ST5.** Cytokine levels of tobacco smoking and biomass-burning smoke groups.

| **Cytokine (pg/mL)** | **COPD-TS**  **(n=40)** | **SWOC**  **(n=40)** | ***p**** | **COPD-BBS**  **(n=40)** | **BBES**  **(n=40)** | ***p**** |
| --- | --- | --- | --- | --- | --- | --- |
| IL-4 | 102.9 (±21.1) | 74.5 (±10.5) | <0.001^a^ | 28.6 (25.2 – 32.2) | 20.2 (17.9 – 22.7) | <0.001 |
| IL-5 | 55.1 (39.2 – 62.3) | 35.7 (30.1 – 43.7) | <0.001 | 48.5 (38.8 – 53.05) | 29.4 (24.7 – 33.1) | <0.001 |
| IL-6 | 18.9 (17.3 – 22.0) | 13.7 (12.4 – 16.8) | <0.001 | 28.3 (23.9 – 32.1) | 19.5 (16.9 – 22.3) | <0.001 |
| IL-10 | 17.4 (±3.9) | 16.9 (±2.7) | 0.528^a^ | 20.8 (19.6 – 24.0) | 22 (19.9 – 24.4) | 0.856 |
| IFN-γ | 13.9 (12.1 – 17.3) | 13.9 (12.6 – 15.6) | 0.421 | 35.6 (±6.5) | 17.6 (±3.3) | <0.001^a^ |

Data are expressed in mean (±standard deviation) or median (quartile 25 – 75). The *p*-values were determined by the Mann-Whitney U test; ^a^ *p-*value was determined by Student’s *t-*test. *Bonferroni correction. Abbreviations: COPD-TS, patients with COPD secondary to tobacco smoking; SWOC, smokers without COPD; COPD-BBS, patients with COPD secondary to biomass-burning smoke; BBES, subjects with chronic exposure to biomass-burning smoke without COPD.

**ST6.** Cytokine levels between genotypes in tobacco smoking and biomass-burning smoke groups, stratified by genotype rs4986790

| **Cytokine (pg/mL)** | **COPD-TS**  **AA**  **(n=30)** | **COPD-TS**  **AG+GG**  **(n=10)** | ***p**** | **SWOC**  **AA**  **(n=19)** | **SWOC**  **AG+GG**  **(n=21)** | ***p**** |
| --- | --- | --- | --- | --- | --- | --- |
| IL-4 | 111.2 (87.9 - 120) | 94.2 (75.7 – 102.7) | 0.033 | 81.2 (76.6 – 90) | 67.8 (65.2 – 72.7) | 0.035 |
| IL-5 | 57.05 (49.07 – 63.6) | 34.9 (29.4 – 40.5) | <0.001 | 44.1 (38.9 – 49.2) | 30.2 (28.2 – 35.1) | <0.001 |
| IL-6 | 18.7 (16.2 – 22.2) | 19.1 (17.4 – 21.4) | 1.0 | 12.8 (12.1 – 16.7) | 14.8 (12.6 – 17.2) | 1.0 |
| IL-10 | 17.9 (±3.9) | 15.9 (±3.4) | 0.626^a^ | 16.4 (±2.9) | 17.4 (±2.6) | 1.0^a^ |
| IFN-γ | 15.6 (12.6 – 18.1) | 12.5 (10.5 – 13.5) | 0.004 | 14.5 (12.6 – 16.3) | 13.6 (12.4 – 15.3) | 1.0 |
| **Cytokine (pg/mL)** | **COPD-BBS**  **AA**  **(n=39)** | **COPD-BBS**  **AG+GG**  **(n=1)** | ***p**** | **BBES**  **AA**  **(n=31)** | **BBES**  **AG+GG**  **(n=9)** | ***p*** |
| IL-4 | 28.8 (26.2 – 32.4) | 18.8 | - | 20.4 (18.4 – 22.8) | 18.3 (16.5 – 22.6) | 0.683 |
| IL-5 | 49.2 (38.9 – 53.2) | 18 | - | 30 (23.4 – 33.8) | 23.7 (20.3 – 27.2) | 0.015 |
| IL-6 | 28.3 (24.7 – 32.1) | 16.2 | - | 18.9 (16.8 – 22.4) | 21.6 (16.8 – 22.3) | 1.0 |
| IL-10 | 20.8 (19.6 – 24.2) | 17.7 | - | 22.4 (19.9 – 25) | 20.5 (18.3 – 22.4) | 0.180 |
| IFN-γ | 36.1 (±6.02) | 19.2 | - | 18.3 (±3.2) | 15.2 (±2.3) | 0.280^a^ |

Data are expressed in mean (±standard deviation) or median (quartile 25 – 75). *p*-values were determined by the Mann-Whitney U test; ^a^ *p-*value was determined by Student’s *t-*test. *Bonferroni correction. Abbreviations: COPD-TS, subjects with COPD secondary to tobacco smoking; SWOC, smokers without COPD; COPD-BBS, subjects with COPD secondary to biomass-burning smoke; BBES, subjects with chronic exposure to biomass-burning smoke without COPD.

| 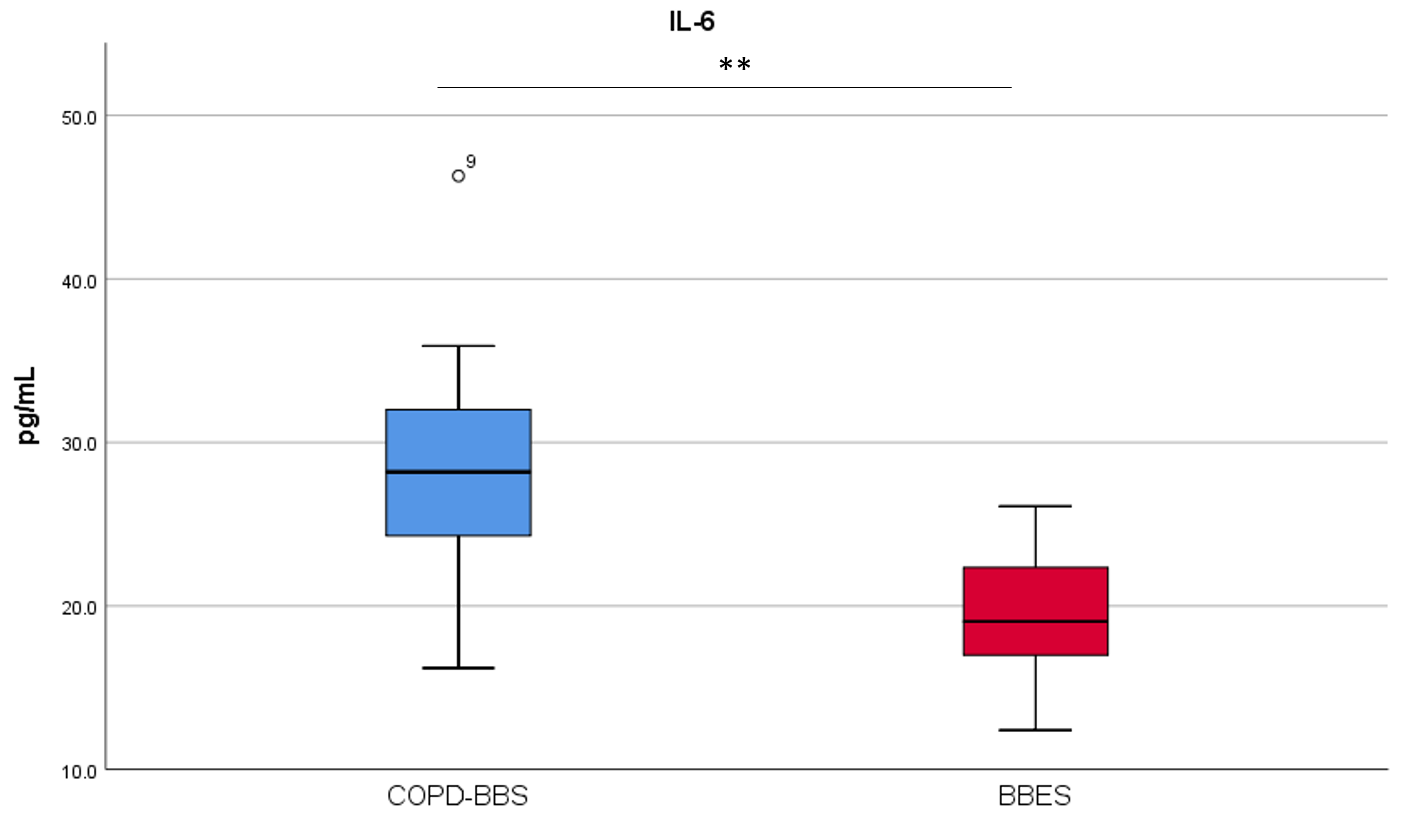 | 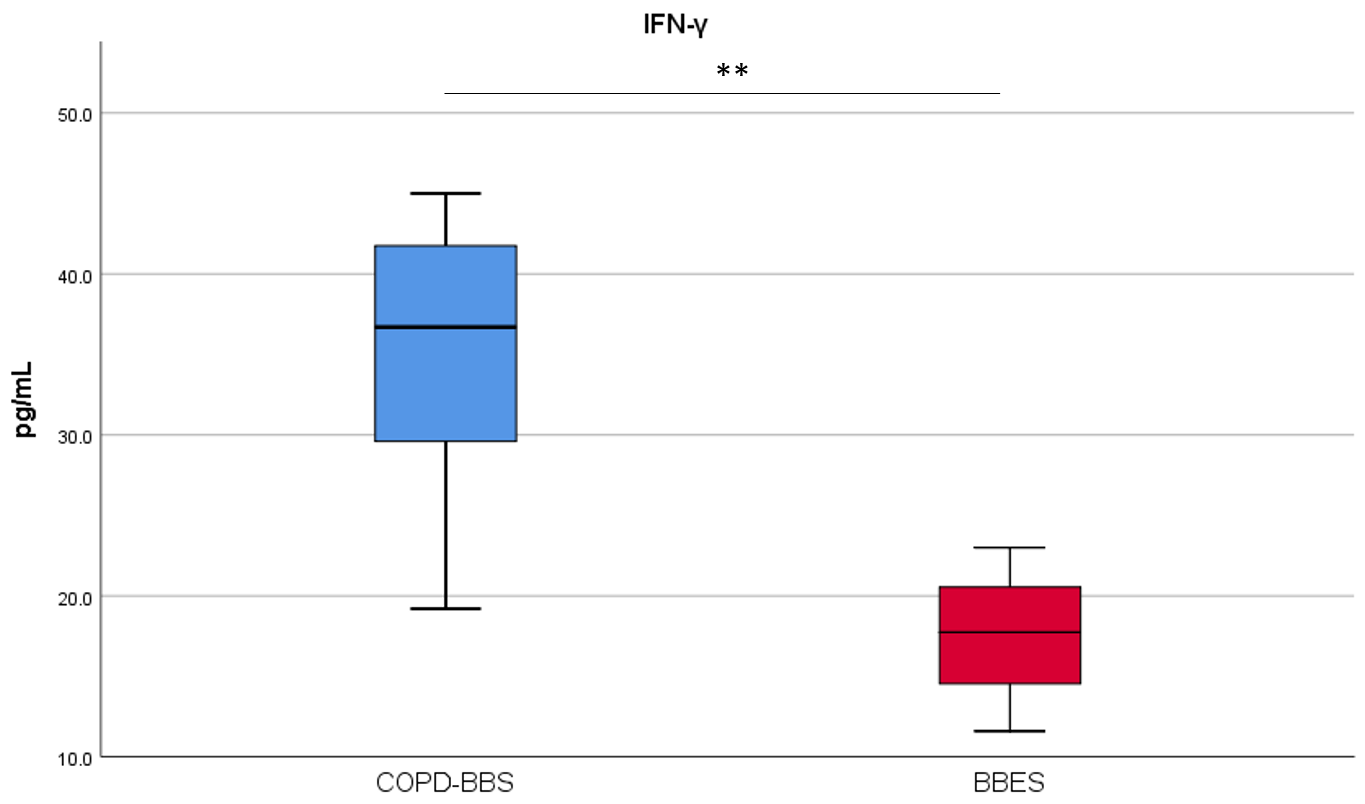 |
| --- | --- |
| (**a**) | (**b**) |
| 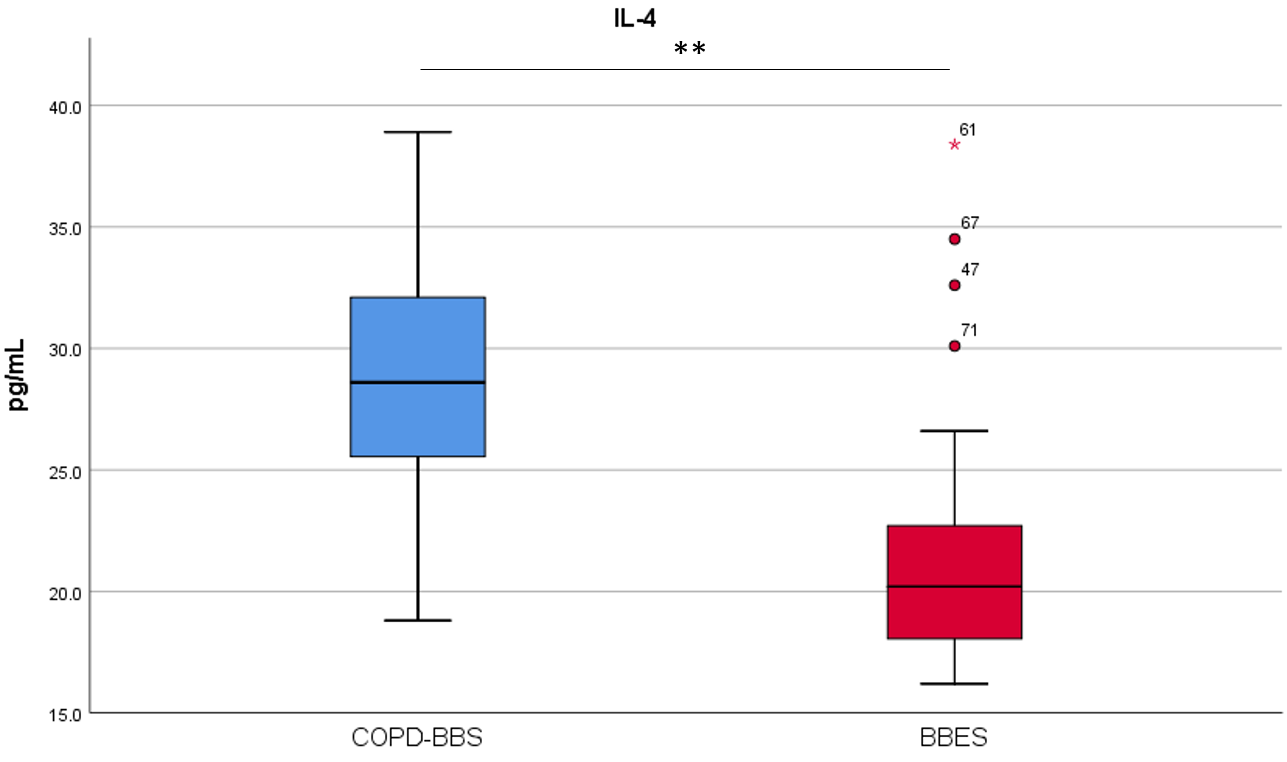 | 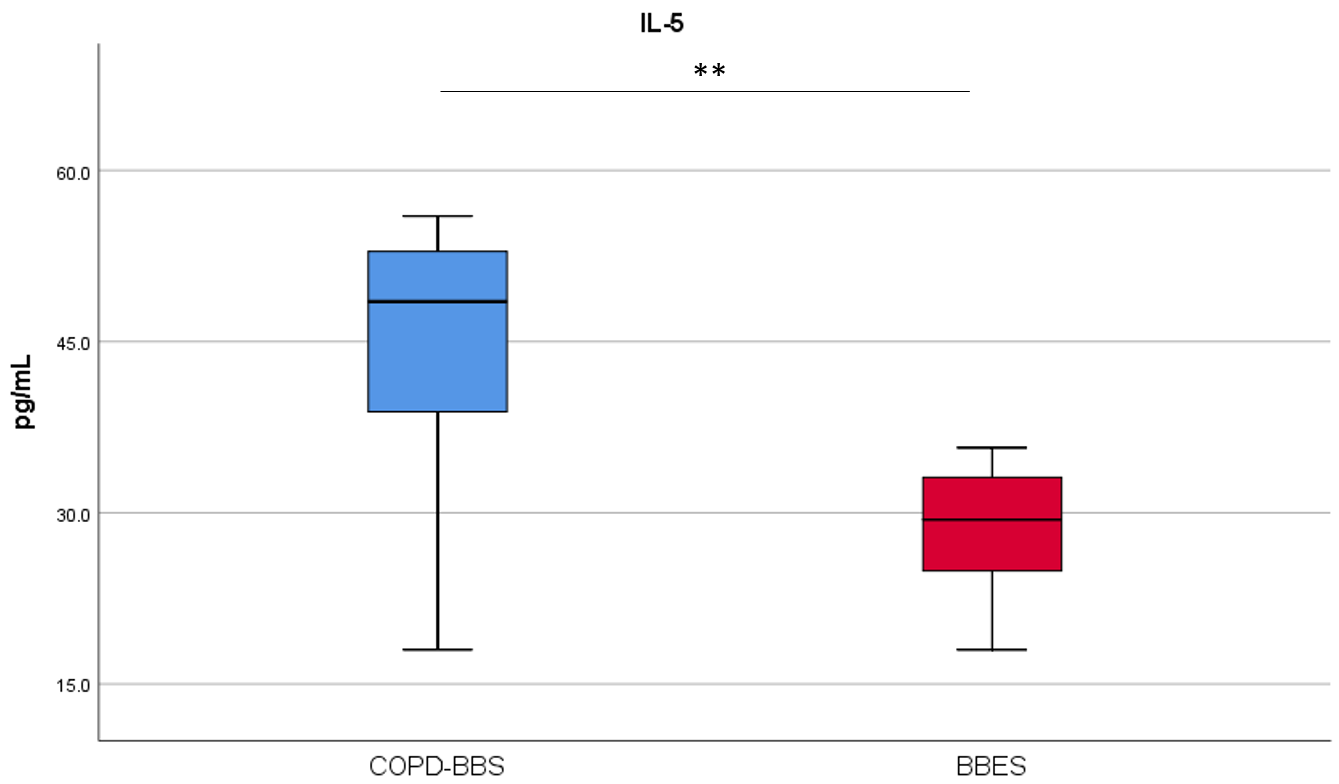 |
| (c) | (d) |
| 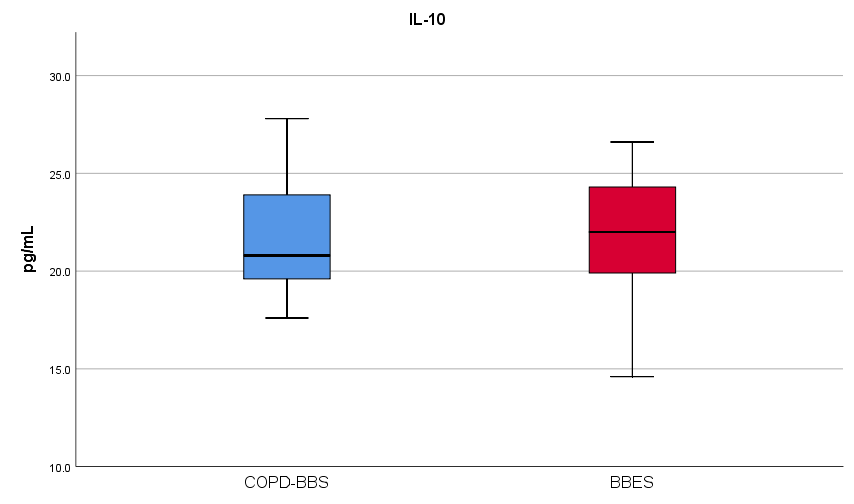 |  |
| (e) |  |

**Supplementary Figure 1 SF1.** Comparison between COPD-BBS (n=40; blue) cytokine levels and BBES (n=40; red). The analysis was performed using the Mann-Whitney U or Student’s *t*-test, depending on the distribution. ** *p*<0.001; * *p*<0.05. (**a**) IL-6; (**b**) IFN-γ; (**c**)IL-4; (**d**) IL-5; (**e**) IL-10. Abbreviations: COPD-BBS, subjects with COPD secondary to biomass-burning smoke; BBES, subjects with chronic exposure to biomass-burning smoke without COPD.
